# Obstetric outcomes in pregnant women with seizure disorder: A hospital-based, longitudinal study

**DOI:** 10.4274/tjod.galenos.2020.87300

**Published:** 2020-10-02

**Authors:** Tarang Preet Kaur, Latika Sahu, Asmita M. Rathore, Sangeeta Bhasin

**Affiliations:** 1 All India Institute of Medical Sciences, Department of Obstetrics and Gynaecology, New Delhi, India; 2 Maulana Azad Medical College, Department of Obstetrics and Gynaecology, New Delhi, India

**Keywords:** Seizure disorder, anti-epileptic drugs, maternal and perinatal complications

## Abstract

**Objective:**

To study the association of seizure disorder with adverse obstetric outcome in terms of maternal and perinatal complications.

**Materials and Methods:**

This longitudinal study was conducted at Maulana Azad Medical College, New Delhi over 15 months among women attending the antenatal clinic (ANC) outpatient department. Fifty pregnant women with seizure disorder with their first ANC visit before 28 weeks were recruited as the case group, excluding patients with eclampsia. The control group included 120 matched healthy pregnant women. After obtaining informed consent, subjects were recruited and followed till one week postpartum and obstetric outcomes were analyzed.

**Results:**

Women with seizure disorder had significantly increased incidence of severe preeclampsia (cases =8%, controls =0%, p<0.001), antepartum hemorrhage (cases =4%, controls =0%, p<0.001), babies with early neonatal complications such as asphyxia (cases =4.1%, controls =0.5%, p=0.04), respiratory distress (cases =14.5%, controls =5.1%, p=0.02), necrotizing enterocolitis (cases =2.0%, controls =0%, p=0.04), early neonatal death (cases =2.0%, controls =0%, p=0.04) and Neonatal Intensive Care Unit admission (cases =20.8%, controls =8.6%, p<0.001) when compared with women without seizure disorder. No significant difference was observed in rates of induction of labor, cesarean section, abortion, congenital anomalies in babies, still births. Conclusion: Women with seizure disorder are at higher risk of hypertensive disorders, antepartum hemorrhage, and early neonatal complications.

**Conclusion:**

Women with seizure disorder are at higher risk of hypertensive disorders, antepartum hemorrhage, and early neonatal complications. Appropriate obstetric, pediatric and neurology care is required during preconception, pregnancy, labor, delivery, and postpartum.

**PRECIS:** Women with seizure disorder are at higher risk of hypertensive disorders, antepartum haemorrhage and early neonatal complications.

## Introduction

Seizure disorder is characterized by an episode of abnormal, uncontrolled neuronal activity in the brain resulting in various manifestations ranging from dramatic convulsive activity, altered consciousness, abnormal sensations to experiential phenomena not readily discernible by an observer^(1)^. Epilepsy as described by International League Against Epilepsy (ILAE) 2014 includes at least 2 unprovoked (or reflex) seizures occurring more than 24 hours apart^(2)^. It is the second most common neurologic problem encountered in pregnancy after headaches with a prevalence of 0.3-0.7%^(3)^.

Women with Epilepsy (WWE) are considered at high risk in pregnancy with maternal mortality 10 times higher than women without seizure disorder^(4)^. Although 90% of WWE have uneventful pregnancies, many studies have shown its association with increased complications^(5)^. Individual studies provide inconclusive estimates of the association between seizure disorder and pregnancy complications such as antenatal [miscarriage, gestational hypertension, preeclampsia, gestational diabetes mellitus (GDM), antepartum hemorrhage (APH), preterm delivery, fetal growth restriction (FGR)], intranatal (need for induction of labor, cesarean section), postnatal [postpartum hemorrhage (PPH)], fetal [low-birth-weight (LBW) babies, need for Neonatal Intensive Care Unit (NICU) admission, congenital fetal anomalies, still births]^(6,7,8,9)^.

Newborns exposed to anti-epileptic drugs (AEDs) in utero have been shown to have 2-3-fold increased (3.3-9%) prevalence of major congenital abnormalities compared with unexposed newborns^(10)^.

Commonly seen congenital malformations include cleft lip and palate, cardiac defects, neural tube defects, skeletal abnormalities, and hypospadias. These malformations are observed more frequently with increased doses of AEDs, higher serum levels of AEDs, and polytherapeutic approaches^(6)^. Folic acid deficiency may also be responsible for the occurrence of major complications to some degree as folic acid supplementation in women under anticonvulsant medications has been shown to decrease malformation rates^(11)^.

Approximately two-thirds of patients experience no change or decrease in the frequency of seizure episodes, whereas the remaining one-third have an increase in frequency. Some of the changes can be attributed to pregnancy-associated physiologic changes and psychological stress. Deliberate patient noncompliance secondary to the fear of the effect of AEDs on the fetus is more likely to be blamed for the increased seizure frequency. It has been seen that lower the number of seizures occurring in the 9 months before conception, the lower the risk of experiencing seizure episodes during pregnancy^(12)^.

Therefore, as the outcomes of pregnant women with seizure disorder is conflicting, we aimed to study the obstetric outcomes of women with seizure disorder and establish whether pregnant such women had an increased risk of complications compared with a control group of matched healthy pregnant women.

### Aims and Objectives

1.    To study the profile of pregnant women with seizure disorder.

2.    To study the obstetric outcome in women with seizure disorder in terms of maternal and perinatal outcomes.

3.    Compare the outcomes with a control group comprising healthy pregnant women.

## Materials and Methods

This was a longitudinal study conducted after obtaining clearance from the Institutional Ethics Committee among women attending the antenatal outpatient department (OPD)/high-risk OPD/gyne casualty in the department of Obstetrics and Gynecology, Maulana Azad Medical College and associated Lok Nayak Hospital, Govind Ballabh Pant Institute of Postgraduate Medical Education and Research (GIPMER) hospital, New Delhi, from November 2016 to January 2017 (15 months) (approval number: 113, date: 04/11/2016). It included 250 pregnant women, out of which 50 women were cases and 200 controls as per the following criteria:

### Selection of Cases

Inclusion criteria: Pregnant women with known case of seizure disorder with first antenatal clinic (ANC) visit between 18 to 28 weeks of gestation.

Exclusion criteria: Pregnant women with eclampsia at the time of enrolment.

- Selection of controls: Age, body mass index (BMI), and gestational age-matched healthy pregnant women. Pregnant women with known medical problems (e.g. chronic hypertension, pre-existing diabetes, asthma, pre-existing hypothyroidism) were excluded.

- Primary outcome: To study obstetric outcomes in women with seizure disorder when compared with women without seizure disorder in terms of maternal complications such as antenatal (abortion, gestational hypertension, preeclampsia, GDM, hypothyroidism, intrahepatic cholestasis of pregnancy, APH, preterm delivery), intranatal (need for induction of labor, caesarean section), postnatal (PPH), fetal (FGR, LBW babies, NICU admissions, congenital fetal anomalies, still births, early neonatal complications such as respiratory distress related, asphyxia related, intraventricular hemorrhage, necrotizing enterocolitis, neonatal hypoglycemia, and 1 and 5-minute Apgar scores).

After obtaining written informed consent from the subjects, data were collected and recorded on a questionnaire. It included baseline demographic details such as age, education status, weight at first visit, height, BMI, significant obstetric history, menstrual history, significant past and family history, pre-pregnancy and trimester-wise intake of folic acid, and preconception counselling.

Detailed history of seizure was recorded in known cases such as time since diagnosis of seizure disorder, seizure type as per the ILAE 2017 classification by a neurologist, underlying cause, date of last seizure prior to pregnancy, seizure episodes during pregnancy and postpartum period, AED intake prior to pregnancy, AEDs consumed during pregnancy, any change in the antiepileptic medication.

For quantifying change in seizure episodes, the frequency in the pre-pregnancy year was taken as the baseline. Increased seizure frequency was defined as a ≥50% increase in the number of seizure episodes from baseline during pregnancy and postpartum period. Decreased seizure frequency was defined as a ≥50% decrease in the number of seizure episodes from baseline during pregnancy and the postpartum period. Any change in seizure frequency between these was defined as unchanged. Abortion is defined as a clinically recognized pregnancy loss before the 20^th^ week of gestation. The World Health Organization defines abortion as expulsion or extraction of an embryo or fetus weighing 500 g or less. Different countries have their own laws for defining criteria for abortion^(13)^. In India, this has been fixed administratively at 28 weeks, when the fetus weighs approximately 1000 g^(14)^. So, any delivery before 28 weeks was categorized as abortion.

Subjects were diagnosed as having gestational hypertension when either systolic blood pressure (BP) was 140 mm Hg or greater or diastolic BP was 90 mm Hg or greater or both, in at least two recordings, at least 4 hours apart^(15)^. Preeclampsia was also defined using criteria by the Task Force on Hypertension in Pregnancy under the American College of Obstetrician and Gynecologists.

To screen and diagnose GDM, the 75 g oral glucose tolerance test as was performed recommended by American Diabetic Association and International Association of Diabetes and Pregnancy Study Groups at first ANC visit for high-risk women or at 24-28 weeks. The cut-offs were taken as follows: fasting blood glucose ≥92 mg/dL, postprandial 1 hour ≥180 mg/dL, postprandial 2 hours ≥153 mg/dL. One or more abnormal values lead to the diagnosis of GDM^(16)^.

Hypothyroidism was defined as per the gestational age specific cut-off for thyroid stimulating hormone, as recommended by American Thyroid Association^(17)^.

FGR was defined as a baby with estimated fetal weight (EFW) or abdominal circumference (AC) less than 10^th^ centile, and severe FGR as EFW AC less than 3^rd^ centile. Fetuses with growth restriction were identified using INTERGROWTH-21^st^ charts^(18)^.

Fetal birth weight was recorded and was further classified as normal (2.5-4.2 kg), LBW (less than 2.5 kg) or large baby (more than 4.2 kg)^(19)^. Apgar scores at 1 and 5 minutes were recorded by a pediatrician.

As per the protocol, all newborns received a 1 mg injection of vitamin K at delivery^(20)^.

Fetal malformations and early neonatal problems were recorded by a pediatrician. They were classified as major or minor malformations. Major congenital anomalies were defined as structural changes that had significant medical, social or cosmetic consequences for the affected individual, and typically require medical intervention. Examples include cleft lip, spina bifida, gastroschisis, meningocele. Minor congenital anomalies were defined as structural changes that posed no significant health problems in the neonatal period and tended to have limited social or cosmetic consequences for the affected individual. Examples include single palmar crease and clinodactyly^(21)^.

Follow-up: Patients were followed up from their first ANC visit till 1 week after delivery to look for early neonatal complications and postpartum seizure episodes.

### Statistical Analysis

Data were analyzed and statistically evaluated using the Statistical Package for the Social Sciences version- Personal Computer (SPSS-PC-17) software.

Quantitative data are expressed in mean, standard deviation, and differences between two comparable groups were tested using Student’s t-test (unpaired) or the Mann-Whitney U test. Qualitative data are expressed as percentages. Statistical differences between the proportions were tested using the chi-square test or Fisher’s Exact test. P-values less than 0.05 were considered statistically significant. Further, odds ratio (OR) and 95% confidence intervals (CI) were used to quantify the risk factors. Univariate analysis was performed, and factors that were found to be significant with p*-*values ≤0.1 were entered in multivariate analysis.

## Results

The baseline demographic data of the study population are shown in Table 1. Preconception counselling was attended by a significantly (p<0.01) greater number of women with seizure disorder (24%) as compared with the controls (2%). Periconception folic acid intake was also observed to be significantly higher (p<0.01) among cases as compared with controls as depicted in the Table 1.

It was also seen that the women with seizure disorder (63%) had a significant (p<0.01) history of abortions in the past when compared with healthy pregnant women (28.5%).

As depicted in Table 2, generalized onset seizure disorder (n=44, 88%) was the most common type seen. Most of the WWE (n=48, 96%) had motor type of seizures and tonic-clonic was the commonest (n=42, 84%) subtype.

Around 46% (n=23) had a seizure disorder of duration between 1-5 years. Among the 30 (60%) women who experienced seizure episodes during pregnancy, the highest number (n=8, 26.6%) had it during the third trimester. Out of these, one woman was not on any AEDs, seven (24.1%) were not compliant with AED intake, and 22 (75.9%) were regularly taking their medication. History of seizure in previous pregnancy had no significant impact on the occurrence of seizure in the current pregnancy (p*=*0.26).

**Figure 1:**
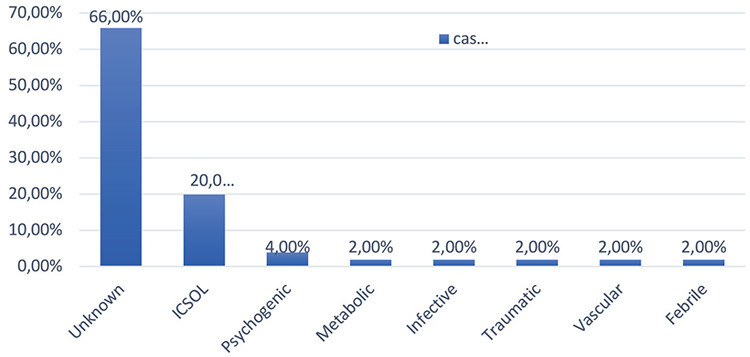
Causes of seizure disorder in cases

The cause of seizure disorder was unknown in 66% (n=33) of the cases. The various causes found are shown in Figure 1.

Out of the 50 women enrolled with seizure disorder, 49 (98%) were taking AEDs. Out of these, six (12%) were started on AEDs during the pregnancy. Most of the women (n=24, 48.9%) were taking second-generation AEDs. Monotherapy was given to 33 (67.2%) women with levetiracetam being the most commonly prescribed AED (n=19, 38.7%).

The most commonly observed change in therapy was the addition of a drug to the pre-existing treatment as seen in 8 (16.3%) women (Table 3).

Women with seizure disorder had a higher incidence of preeclampsia (case n=6, control n=4, p<0.01) and APH (case n=2, control n=0, p<0.01) with no significant difference in the rate of other antenatal complications, as detailed in Table 4. A higher number of women with seizure disorder underwent instrumental vaginal delivery [adjusted odds ratio (aOR)=3.68, CI=1.07-9.64] when compared with the controls. Among the 14 women with seizure disorder who were delivered by cesarean section, two (14.3%) had APH, one (7.1%) had cephalopelvic disproportion, three (21.4%) had failed induction, six (42.8%) had fetal distress, and two (14.3%) had malpresentation.

None of the women with seizure disorder who crossed the period of viability (n=48) had still birth.

Overall, congenital anomalies were observed in three (6.2%) cases and five (2.5%) controls, the difference was not statistically significant. However, minor congenital malformations were significantly (p<0.01) more frequent among the cases (n=2%) than in the controls (0%), as depicted in Table 5. The major congenital malformations among the cases included posterior urethral valve with bilateral hydronephrosis, cleft palate, and polydactyly. Babies with posterior urethral valves had early neonatal demise. Babies born to mothers with seizure disorder had a significantly increased incidence of early neonatal complications (Table 5).

## Discussion

Preconceptional counselling holds an important place in the planning of pregnancy in women with seizure disorder because a careful balance is required between the type, number, and doses of AEDs for the best possible maternal and fetal outcome, thus challenging both the obstetrician and the neurologist.

A significantly higher number of women with seizure disorder went for preconception counselling (case =24%, control =1%, p<0.01) and consumed periconception folic acid (case =30%, control =1%, p<0.01). This reflected increased the awareness created among women with seizure disorder by the referring neurologists at the associated GIPMER hospital regarding preconception counselling and the advantage of intake of folic acid in the prenatal period. Folic acid intake was also documented by Borthen et al.^(3)^ to be significantly greater in WWE than in women without epilepsy (case =43.6%, control =28.5%, p<0.001).

The majority of the cases were on monotherapy (67.3%) with levetiracetam being the most commonly prescribed newer AED (38.7%). Carbamazepine was the second most commonly used AED (14.2%), and the most commonly used old antiepileptic drug. Despite the established risk of teratogenicity with valproate^(10)^. it was consumed by 16.3% of the subjects, either as monotherapy or a part of polytherapy. This could be because our hospital serves as a referral center and most of the time we receive late referrals when women have already taken valproate in periconceptionally and in the early weeks of pregnancy. Here, lies the importance of awareness of preconception counselling and the need for neurologists to avoid valproate in women of reproductive age group wherever possible. In the study by Mawer et al.^(22)^, monotherapy was prescribed to 66.8% of the women, and carbamazepine was the most commonly prescribed drug (26.7%), followed by valproate in 20.6% of subjects. Also, in the study by Ozdemir et al.^(23)^, carbamazepine was the most commonly prescribed AED (27.5%)^(23)^.

The occurrence of seizure episodes was found higher in women with unknown etiology (63.6%) than in women with known etiology (52.9%), but the results did not reach the level of significance (p=0.54) as observed by Chawla and Subbaiah ^(24)^.

It was observed that out of the 30 (60%) women who experienced seizure episodes during pregnancy, 16 (53.3%) had a history of seizure episodes within the 9-month period prior to pregnancy. No significant association (p=0.9) was found between a 9-month seizure-free interval and the prediction of seizure during pregnancy, which was observed in a study by Thomas^(25)^.

The difference in findings could be attributed to their larger sample size.

In this study, seizure frequency was increased in 48% of the subjects, unchanged in 34%, and decreased in 18%. The non-compliance or discontinuation of anticonvulsants might be explained by the women’s concerns about teratogenic effects of these drugs. However, no significant difference was observed in noncompliant patients who did (24.1%) and did not (30%) have seizures during pregnancy (p=0.2). Thus, it could mainly be due to subtherapeutic AED levels occurring due to physiologic alterations in pregnancy leading to increased clearance of the drug^(13)^.

It was also observed that women with seizure disorder had a significantly higher incidence of abortions in previous pregnancies (p<0.001). Both spontaneous and induced abortions were included. It could be the effect of seizure disorder or voluntarily induction out of concern for teratogenicity in the babies or ultrasound findings suggestive of a congenital anomaly.

In this study, 28 (56%) women with seizure disorder developed antenatal complications against 90 (45%) women in the control group, but the difference was not statistically significant (p=0.068). Overall, there was a significantly higher incidence of hypertensive disorders in women with seizure disorder in this study (case =18%, control =7.5%, p=0.02). The rates of gestational hypertension were not significant between the two groups (case =6%, control =5.5%, p=0.92). Preeclampsia with severe features was highly significant in women with seizure disorder in this study (p<0.001) and it was found to be nearly three times more common in women with seizure disorder [adjusted relative risk (aRR)=3.16, CI: 1.67-6.43]. This is similar to the outcome observed by a register-based study on AED-using women where an increased risk of preeclampsia was observed^(26)^. However, the American Academy of Neurology in their report concluded that the evidence was insufficient to support or refute an increased risk of gestational hypertension or preeclampsia in WWE-taking AEDs^(27)^.

APH was significantly higher in women with seizure disorder (p<0.001). Harden et al.^(27)^ also concluded that there was a moderately increased risk of late pregnancy- related bleeding complications. On the other hand, Richmond et al.^(7)^ and Katz et al.^(8)^ reported no increased association of placental abruption in WWE.

No increased association was established in the rates of GDM (p=0.98, aRR=1.06, CI: 0.31-1.89), hypothyroidism (aRR=0.39, CI: 0.04-3.23), intrahepatic cholestasis of pregnancy (aRR=1.17, CI: 0.23-5.86) in women with seizure disorder in this study. Chawla and Subbaiah ^(24)^ also found similar outcomes in their study.

Most of the women with seizure disorder in the present study had a normal vaginal delivery (60.4%). However, this number was significantly less than among healthy pregnant women (73.7%, p=0.01). Risk of instrumental delivery was found to be around 3.7 times higher in women with seizure disorder than in healthy pregnant women. The number of women with seizure disorder who underwent cesarean section were 1.6 times higher than in the controls, but the difference did not reach the level of significance (p=0.06). Harden et al.^(27)^ also concluded a moderately increased risk of cesarean delivery in women with seizure disorder.

LBW babies were born to around 27% mothers with seizure disorder. This rate was slightly higher than among the healthy mothers and was not statistically different (aRR=1.39, CI: 0.66-2.92). Katz et al.^(8)^ also reported no significant association between seizure disorder and LBW babies (p=0.62).

Overall, the incidence of congenital anomalies in this study population was 3.2%. Women with seizure disorder had an incidence of 6% and healthy pregnant women had an incidence of 2.5%. Major congenital anomalies were found in 4% (n=2) of babies born to mothers with seizure disorder and 2.5% (n=5) of the healthy mothers. Out of the three babies of case group with a congenital malformation, two (66.6%) babies were on polytherapy and the mother of one (33.3%) baby was receiving monotherapy with phenytoin. In the polytherapy group, one mother with valproate-based polytherapy gave birth to a baby with polydactyly and the other mother who was on a carbamazepine-based polytherapy had a baby with posterior urethral anomaly.

In the present study, the rates of congenital malformations were 1.4 times higher than in the controls, but it was not statistically significant (aRR=1.4, CI: 0.53-3.06). Borthen et al.^(6)^ found a significantly higher rate of congenital malformations in WWE on anticonvulsive drugs, compared with the control group (p=0.007).

Babies of mothers with seizure disorder in this study were found to have a significantly higher incidence of respiratory distress (p=0.02), necrotizing enterocolitis (p=0.04), asphyxia-related complications (p=0.04), and early neonatal death (p=0.04). The baby who died in the early neonatal period was born preterm, had LBW and also had bilateral posterior urethral valve and went into multiorgan failure. Razaz et al.^(28)^ found an increased risk of neonatal complications in WWE when compared with controls such as hypoglycemia (OR=1.53, CI: 1.34-1.75), infections (OR=1.42, CI: 1.17-1.73), asphyxia-related complications (OR=1.75, CI: 1.26-2.42), and respiratory distress (OR=1.48, CI: 1.30-1.68).

In this present study, admission to the NICU was reported to be 3.4 times more frequent in babies born to women with seizure disorder than in the controls (aRR=3.4, CI: 1.76 -9.35) and was found significant (p=0.01). In a recent meta-analysis by Viale et al.^(29)^, rates of NICU admission were significantly higher in WWE when compared with heathy controls.

Overall, the incidence of congenital anomalies in this study population was 3.2%. Women with seizure disorder had an incidence of 6% and healthy pregnant women had an incidence of 2.5%. Major congenital anomalies were found in 4% (n=2) of babies born to mothers with seizure disorder and 2.5% (n=5) of the healthy mothers. Out of the three babies of case group with a congenital malformation, two (66.6%) babies were on polytherapy and the mother of one (33.3%) baby was receiving monotherapy with phenytoin. In the polytherapy group, one mother with valproate-based polytherapy gave birth to a baby with polydactyly and the other mother who was on a carbamazepine-based polytherapy had a baby with posterior urethral anomaly.

In the present study, the rates of congenital malformations were 1.4 times higher than in the controls, but it was not statistically significant (aRR=1.4, CI: 0.53-3.06). Borthen et al.^(6)^ found a significantly higher rate of congenital malformations in WWE on anticonvulsive drugs, compared with the control group (p=0.007).

The risk of congenital anomalies was not increased in women with seizure disorder. This could be attributed to significantly higher intake of periconceptional folic acid in women with seizure disorder and higher consumption of least teratogenic drugs in this study such as levetiracetam and carbamazepine as was shown by Peterson et al.^(30)^.

### Study Limitations

Large sample size studies are needed to be performed in a prospective manner in order to assess the impact of seizure disorder on maternal and neonatal outcomes. Also, studies need to focus on seizure control in pregnancy, individual AEDs and their dose, and their effect on pregnancy complications.

## Conclusion

Women with seizure disorder and AED use had an increased risk of preeclampsia and APH. The risk of induction of labor and cesarean section was not increased but there was increased risk of instrumental vaginal delivery. Babies of mothers with seizure disorder had an increased risk of early neonatal complications, early neonatal death, and NICU admissions. The risk of congenital anomalies and LBW babies was not increased in women with seizure disorder.

### Interpretation

On the basis of findings of this study, women with seizure disorder should be informed that there is small but significant risk of obstetric complications. Women with seizure disorder should be monitored regularly for BP for the early detection of hypertensive disorders. Attention should be given to women in labor for the early detection and management of APH. Periconception folic acid intake should be encouraged and these women should be managed with the least teratogenic AEDs at the lowest possible dose to diminish the risk of complications, and also maintain good seizure control. Babies born to women with seizure disorder need expert pediatrician follow-up for the management of neonatal complications.

## Figures and Tables

**Table 1:**
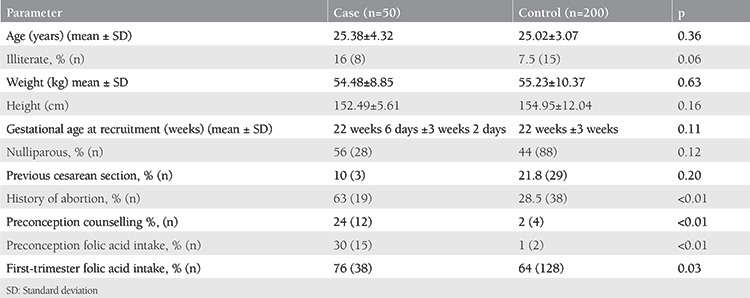
Demographic parameters of subjects

**Table 2:**
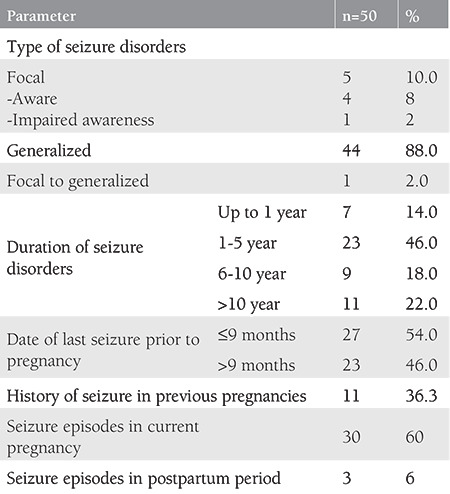
Seizure disorder profile of cases

**Table 3:**
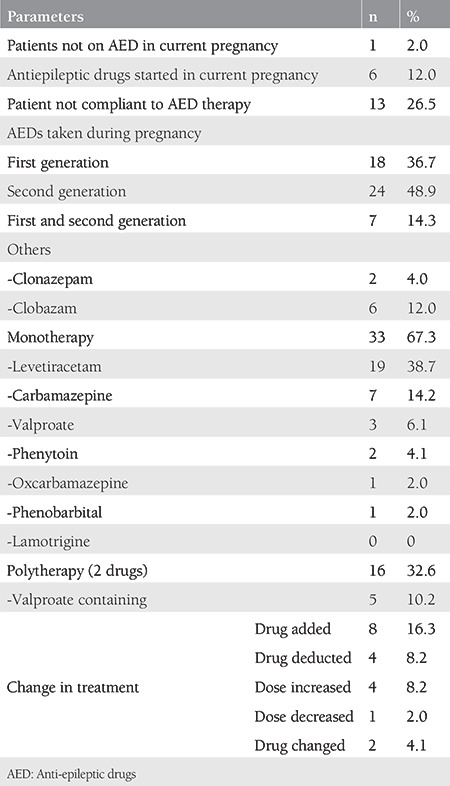
Antiepileptic drug profile of cases

**Table 4:**
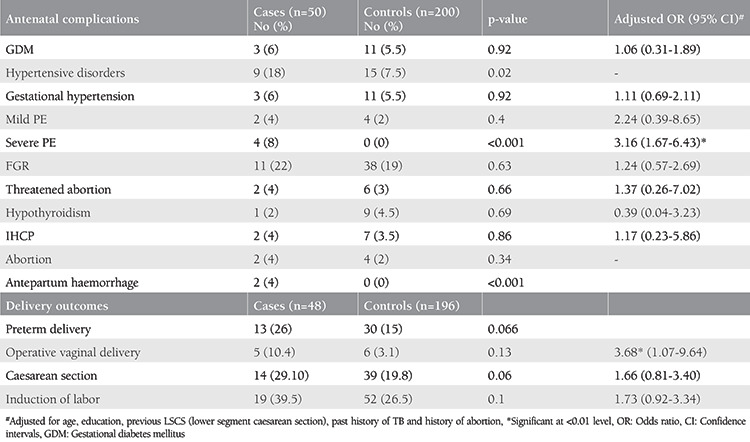
Antenatal complications and delivery outcomes among subjects

**Table 5:**
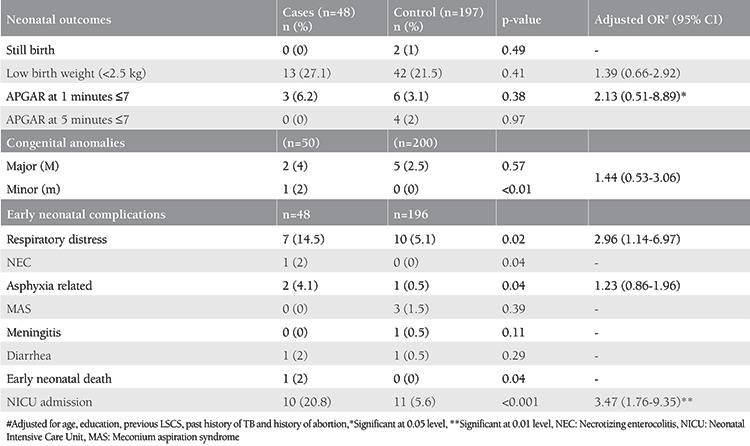
Neonatal outcomes
